# Antioxidant Potential Overviews of Secondary Metabolites (Polyphenols) in Fruits

**DOI:** 10.1155/2020/9081686

**Published:** 2020-05-07

**Authors:** Mohammed Sharif Swallah, He Sun, Raïfatou Affoh, Hongling Fu, Hansong Yu

**Affiliations:** ^1^College of Food Science and Engineering, Jilin Agricultural University, Changchun 130118, China; ^2^College of Economics and Management, Jilin Agricultural University, Changchun 130118, China

## Abstract

The rise in consumption of energy-dense foods has resulted in the displacement of several essential dietary gaps, causing numerous long-lasting diseases, including obesity, stroke, hypertension, and several forms of cancer. Epidemiological studies encourage more fruit consumption to prevent these diseases. The defensive mechanisms provided by these fruits against illness are due to the existence of several antioxidants. Recent studies proved that (poly) phenolic compounds are ideally the core phytochemicals with both functional and health-promoting properties found in the plant's kingdom, and low intake could result in the risk of certain diseases. Phytonutrients are powerful antioxidants that can modify metabolic activation and detoxification of carcinogens. The ideal motive of this review is to provide an overview as well as illuminate the polyphenolic merits of fruits in general. Fruits have several merits, including weight maintenance, proper health development, and satiety. There are many analytical methods for determining and measuring the phenolic content of different products. Phenolic compounds are of nutritional interest since they aid in the retardation and inhibition of lipids by acting as scavengers that prevent and protect the proliferation of oxidative chains. Future studies are required to help identify the physiological metabolic activities as well as to improve human health.

## 1. Introduction

The growth, urbanization, and economic development of Africa and the world at large have resulted in a dietary transition and evolution from a traditional to a modernized diet, where the quality of food has been affected. This transition in today's society has resulted in a high consumption of processed bottled and canned foods with a high content of calories. The rise in the ingestion of these energy-dense foods has caused a high amount of essential nutrients to be displaced in our diets [1]. The consumption of unhealthy diets generates a high nutritional gap, and these are the leading causes of several chronic diseases, including overweight (obesity), cardiovascular diseases, diabetes, stroke, hypertension, and a few types of cancer. Hence, it remains necessary to explore the nutritional merits as well as the constituents of these foodstuffs. In avoidance of these, epidemiological education encourages the intake of more fruits, vegetables, and leguminous plants [2, 3].

For the past years, the consumption rate of fruits has attracted attention since many biochemical and epidemiological studies have focused on the merits associated with the regular intake of these natural diets and the reduction rate of several ailments such as cancer and chronic and heart-related diseases. The defensive mechanisms that these fruits provide against illnesses are associated with the existence of several antioxidants, particularly vitamins (vitamins A, C, E, etc.) and provitamins. Recent studies also indicate plant polyphenolic compounds as the core phytochemicals due to their antioxidant properties [4–7].

**Figure 1:**
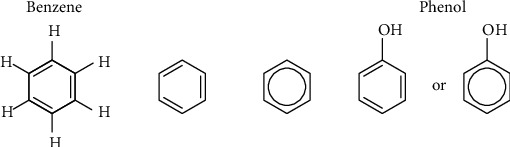
Chemical representation of a phenolic structure.

The term “phenolic” or “polyphenol” is chemically defined as substances that encompass an aromatic ring bearing one or more hydroxyl substituents or groups, with functional by-products (esters, methyl ethers, glycosides, etc.), and their simple molecular structures may differ from composite high-molecular-mass polymer. Thus, “phenol” is a term explaining a phenyl ring having single or extra hydroxyl substituents whiles “polyphenol” is used in expressing natural products having not less than double phenyl rings bearing single or additional hydroxyl substituents. Usually, phenols have double or more hydroxyl groups, which are the biologically active substance that occurs in food plants mostly consumed by a considerable number of people. It is represented chemically as C_6_H_5_OH, with a structure of hydroxyl group (-OH) fused to a phenyl ring. Phenolic compounds comprised of an aromatic benzene ring with single or double hydroxyl groups (e.g., polyphenols) [8–10]. The structure is in Figure 1.

## 2. Phenolic Profile of Fruits and Products

### 2.1. Composition and Classification of Secondary Metabolites

**Figure 2:**
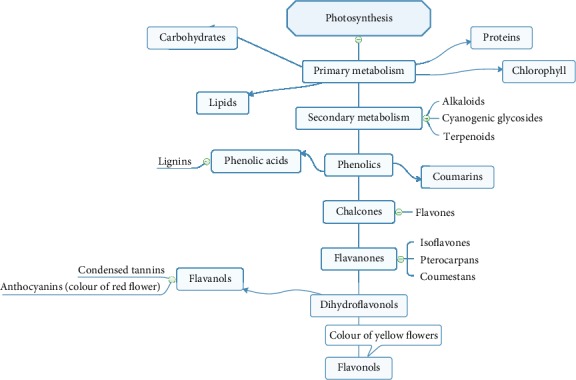
Metabolic flows of polyphenols in plants, adapted from Giada et al. [19].

**Figure 3:**
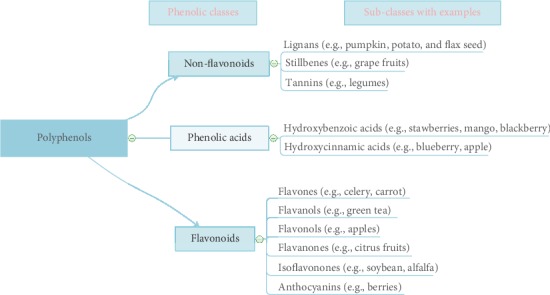
The detail classification of polyphenols, adapted from Basheer and Kerem [20].

Phenolic compounds are considered one of the abundant classes of phytochemicals with health-promoting qualities and functions. Phenolic compounds are classified as secondary metabolites commonly distributed in the kingdom of plants, with immense structures and functions. It is regarded as the most significant and abundant class of compounds in the plant's kingdom. The metabolism of plants is categorized into primary and secondary metabolic forms [11–14]. The primary metabolites include lipids, carbohydrates, proteins, and nucleic acids, as well as all essential elements required for cell growth and development. Secondary metabolites are compounds in specific cells that are indirectly essential for simple respiratory photosynthesis or metabolism but are understood to be vital for a plant's survival. The metabolic flows in plants and detail classification of polyphenol are presented in Figures 2 and 3, respectively [15–20].

Phenolic compounds can be further grouped into water-soluble compounds (phenolic acids, flavonoids, phenylpropanoids, and quinones) and water-insoluble compounds (condensed tannins, lignins, and cell-wall bound hydroxycinnamic acids). This classification is significant due to the nutritional composition or constituents since its solubility and digestibility are needed most for effective utilization within the gastrointestinal tract and some physiological operations. When insoluble phenolic compounds are unable to digest, they will be passed out in the feces wholly or partially, while the soluble compounds can be absorbed through the intestines into the bloodstreams as metabolites. It is subdivided into several forms, e.g., phenolic acids (hydroxybenzoic acids and hydroxycinnamic acids), flavonoids (flavones, flavanols, flavanones, and isoflavones), tannins, stilbenes, and lignans [21–25]. Comparatively, among subphenolic classes, flavonoids are the group with the highest researches due to their supposed health-promoting qualities [26]. Previous studies on cardiovascular diseases were focused mainly on flavones and flavanols, but recent studies have been on nonflavonoid as well as other flavonoid forms [27].

## 3. The Role of Phenolic Compounds in Fruits

Fruits remain a good source of compounds with high phenolic functions, which are leading ingredients in our daily diets [28]. Phenolic compounds, particularly those in fruits, have caught the attention of scientists across the globe, resulting in increased research and reviews in that area [29]. Most of the reports confirm that constant consumption of fruits contributes significantly to a healthy diet, and less consumption could result in the danger of certain chronic diseases like cancer, heart disease, and stroke [30]. Fruits and fruit products play a significant role in human health and diet, predominantly as bases of thiamine, vitamins, niacin, pyridoxine, minerals, folic acid, iron, dietary fiber, magnesium, malic acid, tartaric acid calorie, and citric acids. The phytochemical components (phytonutrients) of some fruits and its products contain high antioxidants, which can alter metabolic activities and cleansing of carcinogens with the potentials of even influencing the factors causing cell tumor [31–33]. There is variation in the phytochemical components of all fruits, thereby resulting in different antioxidant capacity in fruits. Therefore, high consumption leads to high antioxidant capacity. Phyto is a known Greek word denoting plant. Therefore, it is in plants and plant products (vegetables, seeds, grains, legumes, roots, fruits, herbs, nuts, and leaves). Several reports recommend the consumption of at least five serving fruits with vegetables in a day. The nutrients that are possessed by fruits have several merits, including weight maintenance, proper health development, and satiety, as well as supplies a variety of tastes [34, 35].

### 3.1. Phytochemical Components of Some Fruits

Table 1 displays the mean content of some fruits with their phenolic compounds, respectively (mg/100 g of sample) [19].

As displayed in Table 1, phenolic compounds remain generally spread in plant foods (fruits). Blueberry and citrus fruits are predominantly rich with phenolic compounds. Phenolic compounds are the key determinant of the antioxidant potentials in plant foods and thereby represent the fundamental basis of antioxidants.

## 4. Ways of Identifying the Content of Phenolic Compounds in Fruits

**Figure 4:**
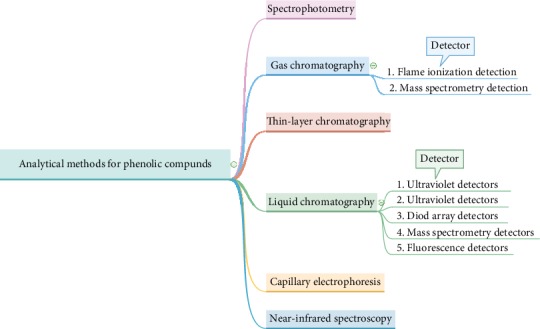
Quantification and classification of analytical methods for phenolic compounds, adapted from Kafkas et al. [37].

Table 1 shows that fruits differ in the amount and even forms of phenolic antioxidants. Therefore, there are many analytical methods available for measuring and determining the sum of phenols in food products, starting with sample preparation methods and extraction per the products (fruits) nature and the phenolic structure [28, 37]. The most vital procedure in the phenolic analysis is both the preparation of samples and the methods used in extraction. Due to the compositional structure of phenolic compounds, it is difficult choosing the best methods for both sample preparation and extractions. Fresh samples (fruits) do not always give better results, even though fresh fruits are the ideal requirement for polyphenolic extraction. This is due to perishability, shelf life, quality, and the season of research. For efficient extraction and preparation of phenolic samples in some situations, it requires either air, shade, or oven drying, lyophilization as well as nitrogen pulverization [38–40], because it solely depends on the type of the medium and the organic composition of samples involved. It includes polarity, molecular structure, concentration, hydroxyl groups, and the number of the aromatic rings involved. Therefore, it is difficult to single out a universal method for the preparation and extraction of phenols for many plant products. Lyophilization is the process used in removing water from materials like food and organic samples. It is regarded as one of the best techniques for prolonging shelf life and product stability [41]. After both preparation of samples and phenolic extraction, it is then followed by classification and quantification with the aid of spectrophotometry, gas chromatography (GC), near-infrared spectroscopy, high-performance liquid chromatography (HPLC), or capillary electrophoresis (CE) methods [42]. The quantification and classification of analytical methods for phenolic compounds are summarized and presented in Figure 4.

### 4.1. Other Tests for Quantification and Separation of Phenolic Compounds

There are other simple but less utilized methods compared to high-performance liquid chromatography and gas chromatography, which can as well aid in the identification of phenolic compounds. These include paper chromatography (PC), high-speed countercurrent chromatography (HSCCC), and supercritical fluid chromatography (SFC) [43].

The SFC is environmentally friendly, with short analysis duration and high separation efficiency, as well as high-resolution power with varied types of detectors. There are a variety of phenolic research done with the aid of SFC techniques which includes polyhydroxy flavonoid identification and extraction of polyphenols in grape seed [43–47]. Paper chromatography (PC) is well used in other studies for the identification of phenolic acids, flavonoids, glycoflavones, and phenolic acids from tea leaf using acetic acid/water/butanol as the mobile phase [48, 49]. High-speed countercurrent chromatography (HSCC) is also known as a biphasic liquid-liquid partitioning method, which is usually used for separation, isolation, and purification of natural compounds [24]. It isolates mixed components or solvent phase due to their partition coefficiency and hydrophobicity. This method uses only liquid samples to ensure the permanent adsorption of the compounds. An efficient and successful separation was achieved using high-speed countercurrent chromatography (HSCC) on a two-phase solvent system consisting of ethyl acetate/methanol/n-hexane/water (15 : 4 : 5 : 7, *v*/*v*) [43].

Several factors influence phenolic extraction, and to achieve successful results requires critical consideration of the following: extraction time, the ratio of both the solute and the solvent, and the size of the sample particles [50].

## 5. The Potential Health Content of (Poly) Phenols in Fruits and Products

### 5.1. What Makes Fruit Consumption Much More Important?

The study on phenolic compounds for the past decades has significantly progressed. The presence of fruit antioxidants is all linked to the health and medicinal merits. Fruits were regarded as high dietary fiber sources and are recently confirmed scientifically to possess essential phytochemicals that are useful and beneficial to human health. Therefore, people who consume fruits as part of their main diet are with the possibility of having a reduced risk of certain chronic diseases [51, 52].

#### 5.1.1. Nutrition, Health Content, and Antioxidant Potentials of Fruits

According to the United States Department of Agriculture (USDA), the most vital nutrients, e.g., potassium, dietary fiber, vitamin C, and foliate, could be obtained from fruits and yet are underconsumed. Naturally, fruits are of low fat, calories, sodium, and zero percent cholesterol. Additionally, diets rich in potassium, folic acid, and vitamin C aid in healthy blood pressure conservation, the formation of red blood cells, and both growth and maintenance of body tissues, respectively. Dietary fiber from fruits helps reduce heart disease risk and blood cholesterol levels [53]. Several fruits have been studied to help identify the hidden molecular mechanisms that will aid in illuminating their health merits. Among all known mechanisms, antioxidant activities possess most phytochemicals [54–62]. Research by Southgate [63] on nature and variability of human food consumption summarized the compositional features of most plant foods, which include fruits, legumes, and vegetables.  Table 2 shows the average compositional features of fruits. Fruits generally contain sugar and fibers, such as pectin, with a high-water content and less amount of fat and protein [63, 64].

Some fruits were studied separately due to their phytochemical contents, which include antioxidants, polyphenols, and phytoestrogens [64]. Phytochemicals can be well-defined as compounds obtained from plants and are considered not to possess any essential nutritional values but have abundant bioactive properties that give environmental protection and encouraging beneficial human health [65, 66]. An antioxidant is any substance that, when available in small quantity or concentration comparatively to an oxidizing substrate, can significantly impede that substrate's oxidation [67]. Therefore, since oxidizing species and other radicals are accountable for oxidative stress resulting in several long-lasting diseases, such as cardiovascular disease, cancer, and diabetes, antioxidant capacity of phytochemicals is the primary mechanism in the prevention of such diseases and promotion of human health [68, 69]. The consumption of this substance or compound is good for human health since it aids in the retardation and inhibition of lipids by acting as scavengers that prevent and protect the proliferation of oxidative chains. Several studies on both humans and animals reveal how polyphenols aid in the inhibition and prevention of cardiovascular diseases as well as cancers when taken on a day-to-day basis. Also, in some cases, they are used purposely due to their pharmaceutical properties [70–74]. For example, polyphenols may intermingle with responsive intermediates and trigger both mutagens and carcinogens, which will aid modulate the actions of the vital proteins which are involved in regulating cell progression cycle as well as influence the activities of various cancer-associated genes [75–78]. It is discovered in Panama that flavanol intake daily is one of the fundamental reasons for recording a decreased incidence of hypertension and cardiovascular diseases. Also, recent analysis supports the fact that polyphenols such as flavanol-rich products reduce blood pressure [79–81]. Further research shows the connections between flavonol, flavanol, and flavone intake and their effect on coronary disease reduction as well as the roles of flavanone and anthocyanin in decreasing cardiovascular disease and mortality [82, 83].

When free radicals are generated within a living organism, several antioxidants provide protection from oxidative damage. This serves as the initial or first line of defense, protective antioxidants, for example, metal-chelating proteins and peroxidases overpower the action and generation of free radicals. Subsequently, the antioxidant radical scavengers, e.g., vitamins C and E, hunt radicals, in order to obstruct the development of oxidative chains and to avoid the spread of chains by serving as the next or second line of defense. It may also include the destroying of chains by the reaction of two radicals. A new enzyme (e.g., proteases, lipases, and transferases) assists by fixing and reconstructing damaged membranes as another line of defense [68, 84].

## 6. Conclusions

In conclusion, plant-based phenolic compounds are one of the vital phytochemicals with a positive response to several disease preventions. Most fruits contain a high number of active antioxidants, and high consumption promotes good health. Primarily, due to its significance and the relationship between consumption and health benefits, there have been vast increments in polyphenol studies. Fruits and beverages, for example, coffee, tea, and red wine, stand as the best and excellent sources of polyphenols, but leguminous plants, vegetables, and cereals are also good sources; therefore, the search for new or improved methodologies for both phenolic extraction and isolation as well as separation, identification, and quantification for various fruits is essential for a clear understanding as well as identifying an excellent source of these phytochemicals. The content of polyphenol in food varies due to genetic variation and technological and environmental factors, which, when altered, will end up affecting the phenolic concentration of food. There is the need to identify plants that are rich in polyphenol, to enhance growing methods and minimize losses during cooking and industrial processing. The mechanism of phenolic absorption is still not well known; therefore, further studies should focus on the potential interactions (metabolism) between polyphenols and the microorganisms within the gastrointestinal tract as well as their physiological activities after administration and upon reaching the site of absorption. Naturally, the phenolic content of fruits, in general, differs thereby resulting in antioxidant variation. Detailed research is needed to help discover the physiological metabolic activities within the living organism as well as to help improve human health by scavenging against human chronic disease-causing organisms.

## Figures and Tables

**Table 1:** Phenolic contents of some fruits.

Fruits	Total phenolics (mg%)
Green apple	118
Red apple	125
Yellow apple	100
Blueberry [36]	327
Sour cherry	156
Sweet cherry	79
Black grape	213
White grape	184
Grape	893
Pink guava	247
White guava	145
Kiwi	791
Lemon	843
Lime	751
Litchi	60
White nectarine	38
Yellow nectarine	25
White peach	53
Yellow peach	35
Pear	125
Pineapple	94
Black plum	88
Red plum	73
Pomegranate	147
Pomelo	57
Raspberry, black	670
Raspberry, red	342
Raspberry, yellow	426
Strawberry	199

Adapted from Giada et al. [19].

**Table 2:** The average compositional features of fruits.

Composition	g/100 g edible matter
Water	61.0–89.1
Protein	0.5–1.1
Fat	Trace–4.4
Sugar	4.4–34.8
Starch	Trace–3.0
Dietary fiber	2.0–14.8
Energy (kcal)	90–646
Micronutrient	Vitamin C, K, Mg, carotenoids
Toxic constituents	Cyanogenic glycosides in seeds

Adapted from Slavin and Lloyd [64].
